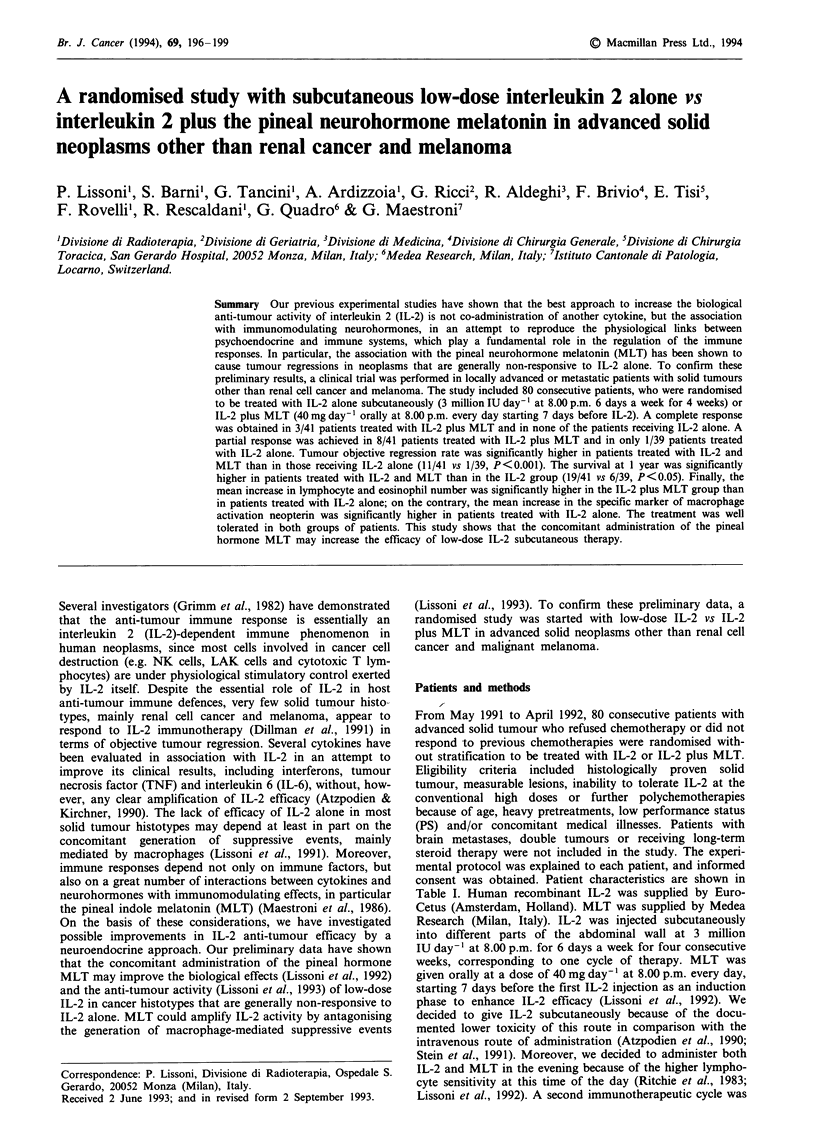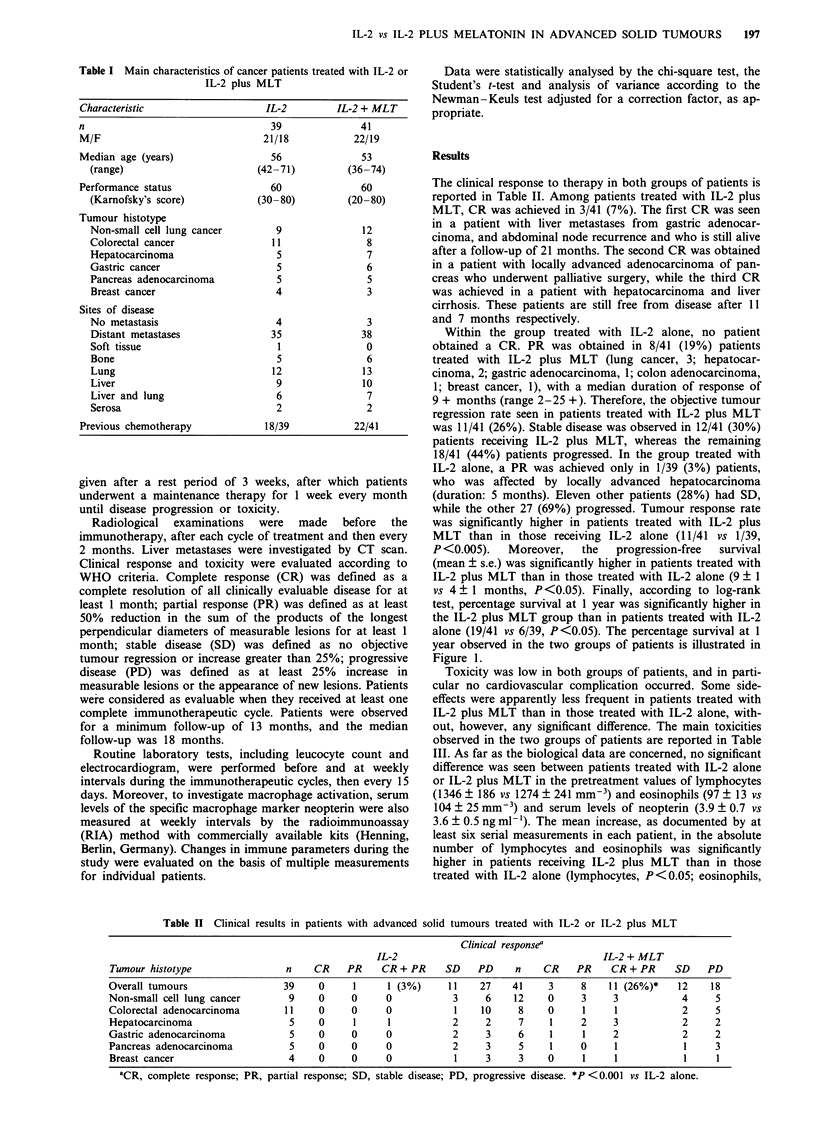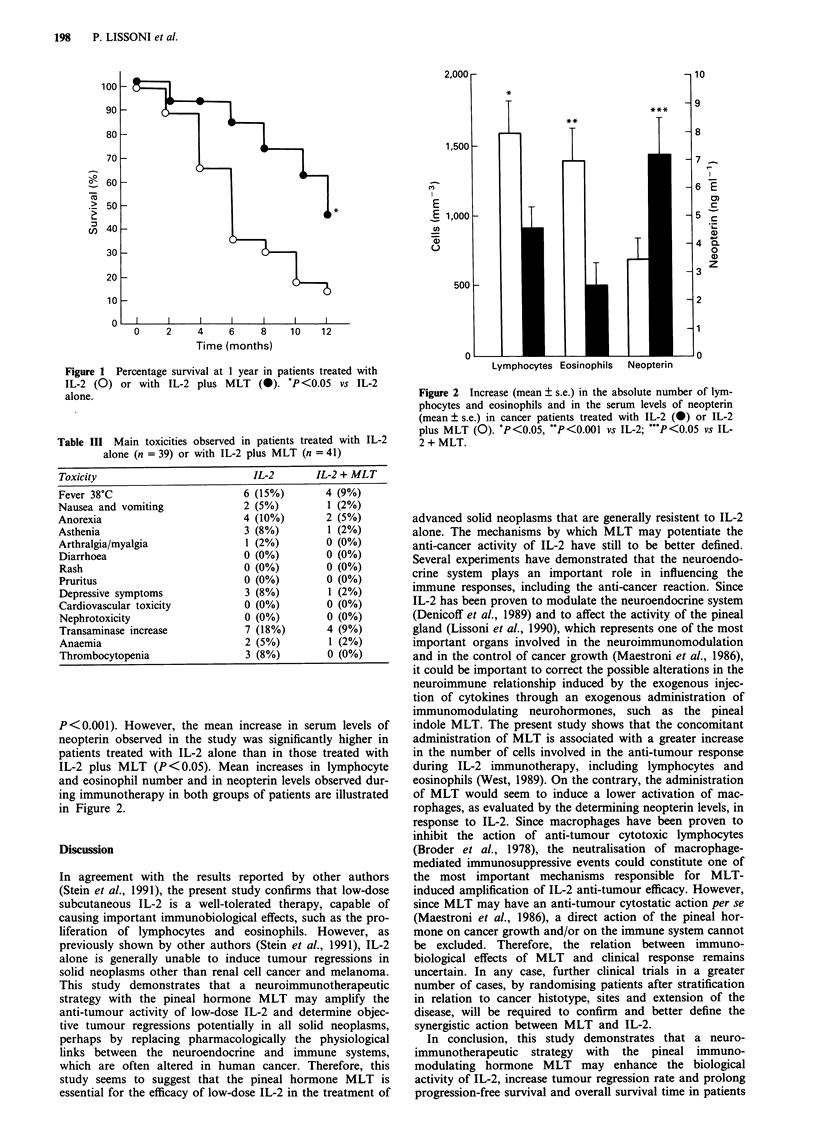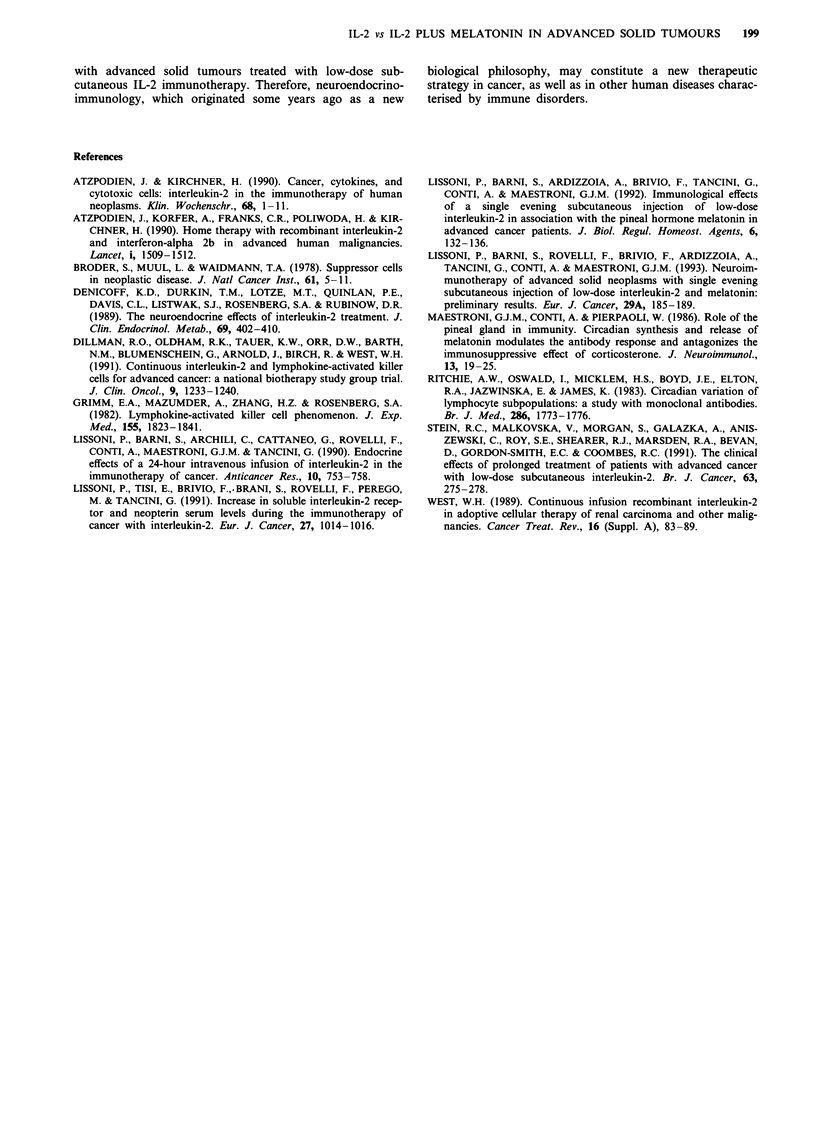# A randomised study with subcutaneous low-dose interleukin 2 alone vs interleukin 2 plus the pineal neurohormone melatonin in advanced solid neoplasms other than renal cancer and melanoma.

**DOI:** 10.1038/bjc.1994.34

**Published:** 1994-01

**Authors:** P. Lissoni, S. Barni, G. Tancini, A. Ardizzoia, G. Ricci, R. Aldeghi, F. Brivio, E. Tisi, F. Rovelli, R. Rescaldani

**Affiliations:** Divisione di Radioterapia, San Gerardo Hospital, Milan, Italy.

## Abstract

Our previous experimental studies have shown that the best approach to increase the biological anti-tumour activity of interleukin 2 (IL-2) is not co-administration of another cytokine, but the association with immunomodulating neurohormones, in an attempt to reproduce the physiological links between psychoendocrine and immune systems, which play a fundamental role in the regulation of the immune responses. In particular, the association with the pineal neurohormone melatonin (MLT) has been shown to cause tumour regressions in neoplasms that are generally non-responsive to IL-2 alone. To confirm these preliminary results, a clinical trial was performed in locally advanced or metastatic patients with solid tumours other than renal cell cancer and melanoma. The study included 80 consecutive patients, who were randomised to be treated with IL-2 alone subcutaneously (3 million IU day-1 at 8.00 p.m. 6 days a week for 4 weeks) or IL-2 plus MLT (40 mg day-1 orally at 8.00 p.m. every day starting 7 days before IL-2). A complete response was obtained in 3/41 patients treated with IL-2 plus MLT and in none of the patients receiving IL-2 alone. A partial response was achieved in 8/41 patients treated with IL-2 plus MLT and in only 1/39 patients treated with IL-2 alone. Tumour objective regression rate was significantly higher in patients treated with IL-2 and MLT than in those receiving IL-2 alone (11/41 vs 1/39, P < 0.001). The survival at 1 year was significantly higher in patients treated with IL-2 and MLT than in the IL-2 group (19/41 vs 6/39, P < 0.05). Finally, the mean increase in lymphocyte and eosinophil number was significantly higher in the IL-2 plus MLT group than in patients treated with IL-2 alone; on the contrary, the mean increase in the specific marker of macrophage activation neopterin was significantly higher in patients treated with IL-2 alone. The treatment was well tolerated in both groups of patients. This study shows that the concomitant administration of the pineal hormone MLT may increase the efficacy of low-dose IL-2 subcutaneous therapy.


Br. J. Cancer (1994), 69, 196-199 © Macmillan Press Ltd., 1994

### A randomised study with subcutaneous low-dose interleukin 2 alone *vs* interleukin 2 plus the pineal neurohormone melatonin in advanced solid neoplasms other than renal cancer and melanoma

P. Lissoni[1], S. Barni[1], G. Tancini[1], A. Ardizzoia[1], G. Ricci[2], R. Aldeghi[3], F. Brivio[4], E. Tisi[5], F. Rovelli[1], R. Rescaldani[1], G. Quadro[6] & G. Maestroni[7]

[1]*Divisione di Radioterapia,* [2]*Divisione di Geriatria,* [3]*Divisione di Medicina,* [4]*Divisione di Chirurgia Generale,* [5]*Divisione di Chirurgia Toracica, San Gerardo Hospital, 20052 Monza, Milan, Italy;* [6]*Medea Research, Milan, Italy;* [7]*Istituto Cantonale di Patologia, Locarno, Switzerland.*

**Summary** Our previous experimental studies have shown that the best approach to increase the biological anti-tumour activity of interleukin 2 (IL-2) is not co-administration of another cytokine, but the association with immunomodulating neurohormones, in an attempt to reproduce the physiological links between psychoendocrine and immune systems, which play a fundamental role in the regulation of the immune responses. In particular, the association with the pineal neurohormone melatonin (MLT) has been shown to cause tumour regressions in neoplasms that are generally non-responsive to IL-2 alone. To confirm these preliminary results, a clinical trial was performed in locally advanced or metastatic patients with solid tumours other than renal cell cancer and melanoma. The study included 80 consecutive patients, who were randomised to be treated with IL-2 alone subcutaneously (3 million IU $day^{-1}$ at 8.00 p.m. 6 days a week for 4 weeks) or IL-2 plus MLT (40 mg $day^{-1}$ orally at 8.00 p.m. every day starting 7 days before IL-2). A complete response was obtained in 3/41 patients treated with IL-2 plus MLT and in none of the patients receiving IL-2 alone. A partial response was achieved in 8/41 patients treated with IL-2 plus MLT and in only 1/39 patients treated with IL-2 alone. Tumour objective regression rate was significantly higher in patients treated with IL-2 and MLT than in those receiving IL-2 alone (11/41 *vs* 1/39, $P<0.001$). The survival at 1 year was significantly higher in patients treated with IL-2 and MLT than in the IL-2 group (19/41 *vs* 6/39, $P<0.05$). Finally, the mean increase in lymphocyte and eosinophil number was significantly higher in the IL-2 plus MLT group than in patients treated with IL-2 alone; on the contrary, the mean increase in the specific marker of macrophage activation neopterin was significantly higher in patients treated with IL-2 alone. The treatment was well tolerated in both groups of patients. This study shows that the concomitant administration of the pineal hormone MLT may increase the efficacy of low-dose IL-2 subcutaneous therapy.

Several investigators (Grimm *et al.*, 1982) have demonstrated that the anti-tumour immune response is essentially an interleukin 2 (IL-2)-dependent immune phenomenon in human neoplasms, since most cells involved in cancer cell destruction (e.g. NK cells, LAK cells and cytotoxic T lymphocytes) are under physiological stimulatory control exerted by IL-2 itself. Despite the essential role of IL-2 in host anti-tumour immune defences, very few solid tumour histotypes, mainly renal cell cancer and melanoma, appear to respond to IL-2 immunotherapy (Dillman *et al.*, 1991) in terms of objective tumour regression. Several cytokines have been evaluated in association with IL-2 in an attempt to improve its clinical results, including interferons, tumour necrosis factor (TNF) and interleukin 6 (IL-6), without, however, any clear amplification of IL-2 efficacy (Atzpodien & Kirchner, 1990). The lack of efficacy of IL-2 alone in most solid tumour histotypes may depend at least in part on the concomitant generation of suppressive events, mainly mediated by macrophages (Lissoni *et al.*, 1991). Moreover, immune responses depend not only on immune factors, but also on a great number of interactions between cytokines and neurohormones with immunomodulating effects, in particular the pineal indole melatonin (MLT) (Maestroni *et al.*, 1986). On the basis of these considerations, we have investigated possible improvements in IL-2 anti-tumour efficacy by a neuroendocrine approach. Our preliminary data have shown that the concomitant administration of the pineal hormone MLT may improve the biological effects (Lissoni *et al.*, 1992) and the anti-tumour activity (Lissoni *et al.*, 1993) of low-dose IL-2 in cancer histotypes that are generally non-responsive to IL-2 alone. MLT could amplify IL-2 activity by antagonising the generation of macrophage-mediated suppressive events (Lissoni *et al.*, 1993). To confirm these preliminary data, a randomised study was started with low-dose IL-2 *vs* IL-2 plus MLT in advanced solid neoplasms other than renal cell cancer and malignant melanoma.

Correspondence: P. Lissoni, Divisione di Radioterapia, Ospedale S. Gerardo, 20052 Monza (Milan), Italy.
Received 2 June 1993; and in revised form 2 September 1993.

### Patients and methods

From May 1991 to April 1992, 80 consecutive patients with advanced solid tumour who refused chemotherapy or did not respond to previous chemotherapies were randomised without stratification to be treated with IL-2 or IL-2 plus MLT. Eligibility criteria included histologically proven solid tumour, measurable lesions, inability to tolerate IL-2 at the conventional high doses or further polychemotherapies because of age, heavy pretreatments, low performance status (PS) and/or concomitant medical illnesses. Patients with brain metastases, double tumours or receiving long-term steroid therapy were not included in the study. The experimental protocol was explained to each patient, and informed consent was obtained. Patient characteristics are shown in Table I. Human recombinant IL-2 was supplied by Euro-Cetus (Amsterdam, Holland). MLT was supplied by Medea Research (Milan, Italy). IL-2 was injected subcutaneously into different parts of the abdominal wall at 3 million IU $day^{-1}$ at 8.00 p.m. for 6 days a week for four consecutive weeks, corresponding to one cycle of therapy. MLT was given orally at a dose of 40 mg $day^{-1}$ at 8.00 p.m. every day, starting 7 days before the first IL-2 injection as an induction phase to enhance IL-2 efficacy (Lissoni *et al.*, 1992). We decided to give IL-2 subcutaneously because of the documented lower toxicity of this route in comparison with the intravenous route of administration (Atzpodien *et al.*, 1990; Stein *et al.*, 1991). Moreover, we decided to administer both IL-2 and MLT in the evening because of the higher lymphocyte sensitivity at this time of the day (Ritchie *et al.*, 1983; Lissoni *et al.*, 1992). A second immunotherapeutic cycle was

**Table I** Main characteristics of cancer patients treated with IL-2 or IL-2 plus MLT

| *Characteristic* | *IL-2* | *IL-2 + MLT* |
|---|---|---|
| *n* | 39 | 41 |
| M/F | 21/18 | 22/19 |
| Median age (years) | 56 | 53 |
| (range) | (42–71) | (36–74) |
| Performance status | 60 | 60 |
| (Karnofsky's score) | (30–80) | (20–80) |
| Tumour histotype | | |
| Non-small cell lung cancer | 9 | 12 |
| Colorectal cancer | 11 | 8 |
| Hepatocarcinoma | 5 | 7 |
| Gastric cancer | 5 | 6 |
| Pancreas adenocarcinoma | 5 | 5 |
| Breast cancer | 4 | 3 |
| Sites of disease | | |
| No metastasis | 4 | 3 |
| Distant metastases | 35 | 38 |
| Soft tissue | 1 | 0 |
| Bone | 5 | 6 |
| Lung | 12 | 13 |
| Liver | 9 | 10 |
| Liver and lung | 6 | 7 |
| Serosa | 2 | 2 |
| Previous chemotherapy | 18/39 | 22/41 |

given after a rest period of 3 weeks, after which patients underwent a maintenance therapy for 1 week every month until disease progression or toxicity.

Radiological examinations were made before the immunotherapy, after each cycle of treatment and then every 2 months. Liver metastases were investigated by CT scan. Clinical response and toxicity were evaluated according to WHO criteria. Complete response (CR) was defined as a complete resolution of all clinically evaluable disease for at least 1 month; partial response (PR) was defined as at least 50% reduction in the sum of the products of the longest perpendicular diameters of measurable lesions for at least 1 month; stable disease (SD) was defined as no objective tumour regression or increase greater than 25%; progressive disease (PD) was defined as at least 25% increase in measurable lesions or the appearance of new lesions. Patients were considered as evaluable when they received at least one complete immunotherapeutic cycle. Patients were observed for a minimum follow-up of 13 months, and the median follow-up was 18 months.

Routine laboratory tests, including leucocyte count and electrocardiogram, were performed before and at weekly intervals during the immunotherapeutic cycles, then every 15 days. Moreover, to investigate macrophage activation, serum levels of the specific macrophage marker neopterin were also measured at weekly intervals by the radioimmunoassay (RIA) method with commercially available kits (Henning, Berlin, Germany). Changes in immune parameters during the study were evaluated on the basis of multiple measurements for individual patients.

Data were statistically analysed by the chi-square test, the Student's *t*-test and analysis of variance according to the Newman–Keuls test adjusted for a correction factor, as appropriate.

### Results

The clinical response to therapy in both groups of patients is reported in Table II. Among patients treated with IL-2 plus MLT, CR was achieved in 3/41 (7%). The first CR was seen in a patient with liver metastases from gastric adenocarcinoma, and abdominal node recurrence and who is still alive after a follow-up of 21 months. The second CR was obtained in a patient with locally advanced adenocarcinoma of pancreas who underwent palliative surgery, while the third CR was achieved in a patient with hepatocarcinoma and liver cirrhosis. These patients are still free from disease after 11 and 7 months respectively.

Within the group treated with IL-2 alone, no patient obtained a CR. PR was obtained in 8/41 (19%) patients treated with IL-2 plus MLT (lung cancer, 3; hepatocarcinoma, 2; gastric adenocarcinoma, 1; colon adenocarcinoma, 1; breast cancer, 1), with a median duration of response of 9 + months (range 2–25 +). Therefore, the objective tumour regression rate seen in patients treated with IL-2 plus MLT was 11/41 (26%). Stable disease was observed in 12/41 (30%) patients receiving IL-2 plus MLT, whereas the remaining 18/41 (44%) patients progressed. In the group treated with IL-2 alone, a PR was achieved only in 1/39 (3%) patients, who was affected by locally advanced hepatocarcinoma (duration: 5 months). Eleven other patients (28%) had SD, while the other 27 (69%) progressed. Tumour response rate was significantly higher in patients treated with IL-2 plus MLT than in those receiving IL-2 alone (11/41 *vs* 1/39, $P<0.005$). Moreover, the progression-free survival (mean ± s.e.) was significantly higher in patients treated with IL-2 plus MLT than in those treated with IL-2 alone (9 ± 1 *vs* 4 ± 1 months, $P<0.05$). Finally, according to log-rank test, percentage survival at 1 year was significantly higher in the IL-2 plus MLT group than in patients treated with IL-2 alone (19/41 *vs* 6/39, $P<0.05$). The percentage survival at 1 year observed in the two groups of patients is illustrated in Figure 1.

Toxicity was low in both groups of patients, and in particular no cardiovascular complication occurred. Some side-effects were apparently less frequent in patients treated with IL-2 plus MLT than in those treated with IL-2 alone, without, however, any significant difference. The main toxicities observed in the two groups of patients are reported in Table III. As far as the biological data are concerned, no significant difference was seen between patients treated with IL-2 alone or IL-2 plus MLT in the pretreatment values of lymphocytes (1346 ± 186 *vs* 1274 ± 241 $mm^{-3}$) and eosinophils (97 ± 13 *vs* 104 ± 25 $mm^{-3}$) and serum levels of neopterin (3.9 ± 0.7 *vs* 3.6 ± 0.5 $ng\,ml^{-1}$). The mean increase, as documented by at least six serial measurements in each patient, in the absolute number of lymphocytes and eosinophils was significantly higher in patients receiving IL-2 plus MLT than in those treated with IL-2 alone (lymphocytes, $P<0.05$; eosinophils,

**Table II** Clinical results in patients with advanced solid tumours treated with IL-2 or IL-2 plus MLT

| | Clinical response[a] | | | | | | | | | | | |
|---|---|---|---|---|---|---|---|---|---|---|---|---|
| | IL-2 | | | | | | IL-2 + MLT | | | | | |
| *Tumour histotype* | *n* | *CR* | *PR* | *CR + PR* | *SD* | *PD* | *n* | *CR* | *PR* | *CR + PR* | *SD* | *PD* |
| Overall tumours | 39 | 0 | 1 | 1 (3%) | 11 | 27 | 41 | 3 | 8 | 11 (26%)* | 12 | 18 |
| Non-small cell lung cancer | 9 | 0 | 0 | 0 | 3 | 6 | 12 | 0 | 3 | 3 | 4 | 5 |
| Colorectal adenocarcinoma | 11 | 0 | 0 | 0 | 1 | 10 | 8 | 0 | 1 | 1 | 2 | 5 |
| Hepatocarcinoma | 5 | 0 | 1 | 1 | 2 | 2 | 7 | 1 | 2 | 3 | 2 | 2 |
| Gastric adenocarcinoma | 5 | 0 | 0 | 0 | 2 | 3 | 6 | 1 | 1 | 2 | 2 | 2 |
| Pancreas adenocarcinoma | 5 | 0 | 0 | 0 | 2 | 3 | 5 | 1 | 0 | 1 | 1 | 3 |
| Breast cancer | 4 | 0 | 0 | 0 | 1 | 3 | 3 | 0 | 1 | 1 | 1 | 1 |

[a]CR, complete response; PR, partial response; SD, stable disease; PD, progressive disease. *$P<0.001$ *vs* IL-2 alone.

Figure 1 Percentage survival at 1 year in patients treated with IL-2 (○) or with IL-2 plus MLT (●). *$P<0.05$ vs IL-2 alone.

Figure 2 Increase (mean ± s.e.) in the absolute number of lymphocytes and eosinophils and in the serum levels of neopterin (mean ± s.e.) in cancer patients treated with IL-2 (●) or IL-2 plus MLT (○). *$P<0.05$, **$P<0.001$ vs IL-2; ***$P<0.05$ vs IL-2 + MLT.

**Table III** Main toxicities observed in patients treated with IL-2 alone ($n$ = 39) or with IL-2 plus MLT ($n$ = 41)

| *Toxicity* | *IL-2* | *IL-2 + MLT* |
|---|---|---|
| Fever 38°C | 6 (15%) | 4 (9%) |
| Nausea and vomiting | 2 (5%) | 1 (2%) |
| Anorexia | 4 (10%) | 2 (5%) |
| Asthenia | 3 (8%) | 1 (2%) |
| Arthralgia/myalgia | 1 (2%) | 0 (0%) |
| Diarrhoea | 0 (0%) | 0 (0%) |
| Rash | 0 (0%) | 0 (0%) |
| Pruritus | 0 (0%) | 0 (0%) |
| Depressive symptoms | 3 (8%) | 1 (2%) |
| Cardiovascular toxicity | 0 (0%) | 0 (0%) |
| Nephrotoxicity | 0 (0%) | 0 (0%) |
| Transaminase increase | 7 (18%) | 4 (9%) |
| Anaemia | 2 (5%) | 1 (2%) |
| Thrombocytopenia | 3 (8%) | 0 (0%) |

$P<0.001$). However, the mean increase in serum levels of neopterin observed in the study was significantly higher in patients treated with IL-2 alone than in those treated with IL-2 plus MLT ($P<0.05$). Mean increases in lymphocyte and eosinophil number and in neopterin levels observed during immunotherapy in both groups of patients are illustrated in Figure 2.

### Discussion

In agreement with the results reported by other authors (Stein *et al.*, 1991), the present study confirms that low-dose subcutaneous IL-2 is a well-tolerated therapy, capable of causing important immunobiological effects, such as the proliferation of lymphocytes and eosinophils. However, as previously shown by other authors (Stein *et al.*, 1991), IL-2 alone is generally unable to induce tumour regressions in solid neoplasms other than renal cell cancer and melanoma. This study demonstrates that a neuroimmunotherapeutic strategy with the pineal hormone MLT may amplify the anti-tumour activity of low-dose IL-2 and determine objective tumour regressions potentially in all solid neoplasms, perhaps by replacing pharmacologically the physiological links between the neuroendocrine and immune systems, which are often altered in human cancer. Therefore, this study seems to suggest that the pineal hormone MLT is essential for the efficacy of low-dose IL-2 in the treatment of advanced solid neoplasms that are generally resistent to IL-2 alone. The mechanisms by which MLT may potentiate the anti-cancer activity of IL-2 have still to be better defined. Several experiments have demonstrated that the neuroendocrine system plays an important role in influencing the immune responses, including the anti-cancer reaction. Since IL-2 has been proven to modulate the neuroendocrine system (Denicoff *et al.*, 1989) and to affect the activity of the pineal gland (Lissoni *et al.*, 1990), which represents one of the most important organs involved in the neuroimmunomodulation and in the control of cancer growth (Maestroni *et al.*, 1986), it could be important to correct the possible alterations in the neuroimmune relationship induced by the exogenous injection of cytokines through an exogenous administration of immunomodulating neurohormones, such as the pineal indole MLT. The present study shows that the concomitant administration of MLT is associated with a greater increase in the number of cells involved in the anti-tumour response during IL-2 immunotherapy, including lymphocytes and eosinophils (West, 1989). On the contrary, the administration of MLT would seem to induce a lower activation of macrophages, as evaluated by the determining neopterin levels, in response to IL-2. Since macrophages have been proven to inhibit the action of anti-tumour cytotoxic lymphocytes (Broder *et al.*, 1978), the neutralisation of macrophage-mediated immunosuppressive events could constitute one of the most important mechanisms responsible for MLT-induced amplification of IL-2 anti-tumour efficacy. However, since MLT may have an anti-tumour cytostatic action *per se* (Maestroni *et al.*, 1986), a direct action of the pineal hormone on cancer growth and/or on the immune system cannot be excluded. Therefore, the relation between immunobiological effects of MLT and clinical response remains uncertain. In any case, further clinical trials in a greater number of cases, by randomising patients after stratification in relation to cancer histotype, sites and extension of the disease, will be required to confirm and better define the synergistic action between MLT and IL-2.

In conclusion, this study demonstrates that a neuroimmunotherapeutic strategy with the pineal immunomodulating hormone MLT may enhance the biological activity of IL-2, increase tumour regression rate and prolong progression-free survival and overall survival time in patients with advanced solid tumours treated with low-dose subcutaneous IL-2 immunotherapy. Therefore, neuroendocrinoimmunology, which originated some years ago as a new biological philosophy, may constitute a new therapeutic strategy in cancer, as well as in other human diseases characterised by immune disorders.

### References

ATZPODIEN, J. & KIRCHNER, H. (1990). Cancer, cytokines, and cytotoxic cells: interleukin-2 in the immunotherapy of human neoplasms. *Klin. Wochenschr.*, **68,** 1-11.

ATZPODIEN, J., KORFER, A., FRANKS, C.R., POLIWODA, H. & KIRCHNER, H. (1990). Home therapy with recombinant interleukin-2 and interferon-alpha 2b in advanced human malignancies. *Lancet*, **i,** 1509-1512.

BRODER, S., MUUL, L. & WAIDMANN, T.A. (1978). Suppressor cells in neoplastic disease. *J. Natl Cancer Inst.*, **61,** 5-11.

DENICOFF, K.D., DURKIN, T.M., LOTZE, M.T., QUINLAN, P.E., DAVIS, C.L., LISTWAK, S.J., ROSENBERG, S.A. & RUBINOW, D.R. (1989). The neuroendocrine effects of interleukin-2 treatment. *J. Clin. Endocrinol. Metab.*, **69,** 402-410.

DILLMAN, R.O., OLDHAM, R.K., TAUER, K.W., ORR, D.W., BARTH, N.M., BLUMENSCHEIN, G., ARNOLD, J., BIRCH, R. & WEST, W.H. (1991). Continuous interleukin-2 and lymphokine-activated killer cells for advanced cancer: a national biotherapy study group trial. *J. Clin. Oncol.*, **9,** 1233-1240.

GRIMM, E.A., MAZUMDER, A., ZHANG, H.Z. & ROSENBERG, S.A. (1982). Lymphokine-activated killer cell phenomenon. *J. Exp. Med.*, **155,** 1823-1841.

LISSONI, P., BARNI, S., ARCHILI, C., CATTANEO, G., ROVELLI, F., CONTI, A., MAESTRONI, G.J.M. & TANCINI, G. (1990). Endocrine effects of a 24-hour intravenous infusion of interleukin-2 in the immunotherapy of cancer. *Anticancer Res.*, **10,** 753-758.

LISSONI, P., TISI, E., BRIVIO, F., BRANI, S., ROVELLI, F., PEREGO, M. & TANCINI, G. (1991). Increase in soluble interleukin-2 receptor and neopterin serum levels during the immunotherapy of cancer with interleukin-2. *Eur. J. Cancer*, **27,** 1014-1016.

LISSONI, P., BARNI, S., ARDIZZOIA, A., BRIVIO, F., TANCINI, G., CONTI, A. & MAESTRONI, G.J.M. (1992). Immunological effects of a single evening subcutaneous injection of low-dose interleukin-2 in association with the pineal hormone melatonin in advanced cancer patients. *J. Biol. Regul. Homeost. Agents*, **6,** 132-136.

LISSONI, P., BARNI, S., ROVELLI, F., BRIVIO, F., ARDIZZOIA, A., TANCINI, G., CONTI, A. & MAESTRONI, G.J.M. (1993). Neuroimmunotherapy of advanced solid neoplasms with single evening subcutaneous injection of low-dose interleukin-2 and melatonin: preliminary results. *Eur. J. Cancer*, **29A,** 185-189.

MAESTRONI, G.J.M., CONTI, A. & PIERPAOLI, W. (1986). Role of the pineal gland in immunity. Circadian synthesis and release of melatonin modulates the antibody response and antagonizes the immunosuppressive effect of corticosterone. *J. Neuroimmunol.*, **13,** 19-25.

RITCHIE, A.W., OSWALD, I., MICKLEM, H.S., BOYD, J.E., ELTON, R.A., JAZWINSKA, E. & JAMES, K. (1983). Circadian variation of lymphocyte subpopulations: a study with monoclonal antibodies. *Br. J. Med.*, **286,** 1773-1776.

STEIN, R.C., MALKOVSKA, V., MORGAN, S., GALAZKA, A., ANISZEWSKI, C., ROY, S.E., SHEARER, R.J., MARSDEN, R.A., BEVAN, D., GORDON-SMITH, E.C. & COOMBES, R.C. (1991). The clinical effects of prolonged treatment of patients with advanced cancer with low-dose subcutaneous interleukin-2. *Br. J. Cancer*, **63,** 275-278.

WEST, W.H. (1989). Continuous infusion recombinant interleukin-2 in adoptive cellular therapy of renal carcinoma and other malignancies. *Cancer Treat. Rev.*, **16** (Suppl. A), 83-89.